# Failure Mode Analysis of Aluminium Alloy 2024-T3 in Double-Lap Bolted Joints with Single and Double Fasteners; A Numerical and Experimental Study

**DOI:** 10.3390/ma8063195

**Published:** 2015-06-03

**Authors:** Khosro Fallahnezhad, Andrew Steele, Reza H. Oskouei

**Affiliations:** Discipline of Mechanical Engineering, School of Computer Science, Engineering and Mathematics, Flinders University, Tonsley 5042, Australia; E-Mails: khosro.fallahnezhad@flinders.edu.au (K.F.); stee0152@flinders.edu.au (A.S.)

**Keywords:** aluminium alloys, mechanically fastened joints, failure mode, finite element analysis

## Abstract

This paper aims to study the mechanical behaviour and failure mode of Al 2024-T3 in double-lap bolted joints. To accomplish this, the effect of geometric parameters was investigated in both configurations of single and double fasteners. Using ABAQUS/Standard, a three-dimensional finite element model was developed and verified against the experimental results of the joints loaded in tension. In general, double bolt joints were found to have greater load carrying capacities than single bolt joints (by 40%–49%). In single bolt joints, the plate width had insignificant effect on the behaviour of the joint under tensile loading; whereas, increasing the distance of the hole from the edge, considerably enhanced the strength of the joint. In double bolt joints, changing the edge distance had almost no effect on the behaviour of the joint. However, increasing the plate width from 25.4 to 30 mm increased the load carrying capacity by 28%. This study showed that in single bolt connections, with increasing the edge distance, the failure mode can favourably shift from shear-out to bearing. Also, double bolt joints with wider plates (increased width) can beneficially shift the failure mode from net-tension to bearing. The geometric parameters were found to play an important role in controlling the failure mode so that catastrophic failure modes of net-tension and shear-out can be prevented in bolted joint.

## 1. Introduction

Mechanical fasteners are largely used in the construction of aircraft. These fasteners form the complex component geometries required for flight, durability, and performance. There have been several investigations on different aspects of mechanically fastened joints such as fastener type, stress concentration, clamping force, and fastening patterns that contribute to the mechanical behaviour of the joints under loading [1,2,3,4]. Drilling holes in joining plates may cause a high stress concentration around the hole and this may decrease the strength of the joints [5,6]. From previous research, it can be understood that clamping force [7,8], cold expansion [6,9] and interference fit [10,11,12,13] can reduce the stress concentration at the hole area; and thus can increase resistance of the joints against fatigue. Finite element (FE) modelling has been used in previous studies to better investigate the mechanical behaviour of bolted and riveted joints. Iyer *et al.* [14] investigated fatigue behaviour of single- and double-rivet self-piercing riveted joints using experimental tests and finite element modelling. Using a three-dimensional elastic finite element model, they located the gross section crack initiation. Armentani *et al.* [15] analysed multiple crack propagation in an aeronautic doubler-skin riveted joint. They implemented both dual boundary element method and finite element method and compared advantages and disadvantages of these two methods. Oskouei *et al.* [16] developed a three-dimensional finite element model to simulate the clamping force and transfer the applied tensile loads through the joint plates. This study revealed that increasing the clamping force can decrease the stress magnitude around the hole under tensile loading. Kim *et al.* [17] proposed four types of finite element models to investigate bolted joint structures. It was concluded that the model with three dimensional solid elements and surface-to-surface contact elements between the bolt head/nut and flange interfaces has the best agreement with the experimental results.

Failure modes of fastened joints have always been important to study in order to improve mechanical design of joined components. Pisano *et al.* [18] numerically investigated the methodology of “limit analysis theory”. This methodology was applied to multi-pin joints composite laminates in order to predict the failure mode of fastenings. Chen *et al.* [19] experimentally and numerically studied failure modes of riveted joints under tensile loading. Generally, they reported three types of failure modes including pull through, shank breaking, and head breaking. Results of simulations were in good agreement with experimental findings. Furthermore, three formulas were proposed to calculate the maximum tensile strength of the riveted joints. Generally, failure modes in bolted joints can be divided into two categories: basic failure modes and secondary failure modes. Net-tension, bearing and shear-out are the three basic failure modes and cleavage and shear-out are secondary failure modes [20,21]. Geometric parameters seem to play a main role in failure modes of bolted joints. Distance of the hole from the edge of the plate and width of the plate are two parameters that can effectively contribute to the type of failure mode [22,23]. There have been limited investigations on failure modes of bolted joints with different configurations. Keikhosravy *et al.* [24] investigated the effect of geometric variables on the stress and strain distributions, as well as non-linear deformation behaviour of aluminium alloy 2024-T3 single-lap bolted joints loaded in tension. They found that in a single-lap bolted joint, increasing the distance of the hole from the plate edge and the plate width can shift the failure mode from net-tension and shear-out to bearing. In a recent study [25], the tensile strength and failure of single shear bolted joints of Al 6061-T6 and 7075-T6 were investigated. The main variables included the plate thickness, end distance and bolt arrangement. Two failure modes of shear fracture and block shear fracture were reported. However, these investigations were limited to single-lap joints, whereas, double-lap joints are more commonly used in structures.

In this paper, mechanical behaviour of double-lap bolted joints made from 2024-T3 aluminium plates was studied. A three-dimensional finite element model was developed using ABAQUS/Standard to investigate both single and double bolt joints with different geometries to better understand the failure mode mechanism in these joints. The model was verified by a set of experimental results. Stress distributions and the effect of main geometric parameters on the mode of failure were studied and discussed.

## 2. Experimental Approach

**Figure 1:**
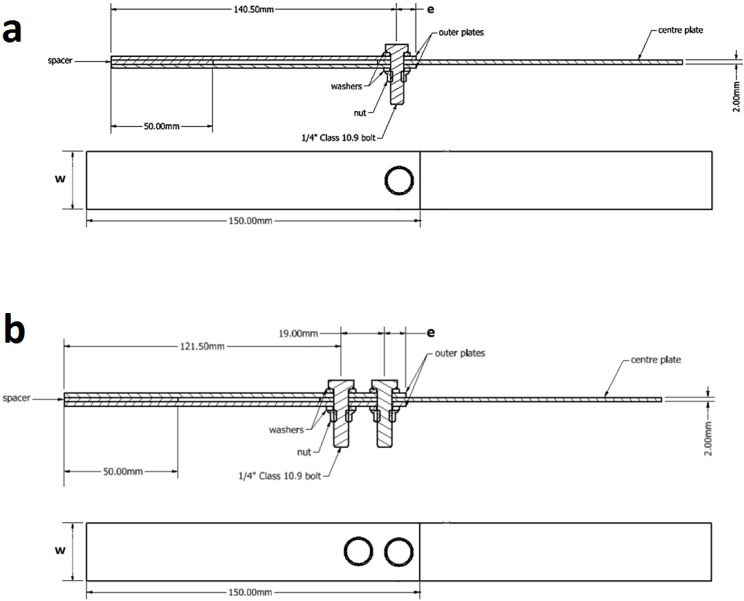
Bolted joint specimens: (**a**) single bolt joint; and (**b**) double bolt joint.

Double-lap joint specimens were configured by fastening three identical plates together through both single bolt and double bolts, as shown in Figure 1. The joint plates were machined from a 2024-T3 alclad aluminium alloy panel such that the longitudinal axis of the plates was parallel to the rolling direction of the panel. The thickness of the plates was t = 2 mm. Circular fastener holes of d = 6.5 mm in diameter were drilled at the centre of the specimen width with free edge distances of e = 9.5 and 12.7 mm. One-quarter inch-diameter high strength steel bolts (class 12.9) were used to clamp the specimen halves and a 15 N·m tightening torque was applied with a digital torque wrench (PRO PLUS Precision, Matador, Germany). The length of the bolt was determined by selecting a bolt with a shank length long enough to protrude through the total joint thickness with two washers (Figure 1).

A number of preliminary tests were performed to ensure applying a high tightening torque without inducing any plastic deformation in the bolt/nut threads. To do this, different tightening torque values with 3 N·m increments were applied to the bolt and nut at the joint. After applying each tightening torque, the bolt and nut were unscrewed and the threads were visually inspected for any evidence of plastic deformation. Also, while undoing the bolt and nut, the smoothness of the nut movement was checked to detect plastic deformation of the threads. Fifteen Newton metre was found to be almost the highest torque while the fasteners were in the elastic region with no plasticity in the threads.

Tensile loads were applied using an Instron universal testing machine (model 5969) capable of generating a maximum tensile load of 50 kN. A quasi-static testing method was developed to apply the tensile load with a displacement rate of 0.3 mm/min to the specimens. Load and displacement data were recorded from the start to the failure point of the test specimens. For each experiment, three tests were conducted and the average of the results was used for analysis. The middle plate, as the critical load carrying plate, was inspected for its failure. A scanning electron microscope (SEM), Aspex PSEM eXpress, was used to examine the features on the material surface both in the bore of the hole and around the hole at high magnifications.

## 3. Finite Element Modelling Approach

Double-lap bolted joint models were developed using ABAQUS 6.13. Identical to the test specimens, the model included three identical plates of aluminium alloy 2024-T3, with a thickness of 2 mm and a 6.5 mm-diameter hole as well as a high strength steel bolt to clamp the plates. Isotropic material properties with elastic behaviour was defined for the steel bolt with an elastic modulus of E = 200 GPa and a Poisson’s ratio of ν = 0.3. Elastic-plastic isotropic material properties with an elastic modulus of E = 72.4 GPa, Poisson’s ratio of ν = 0.33, yield strength of 302 MPa, ultimate tensile strength of 470 MPa and fracture strain of 18.9% were set for the material model of the aluminium plates. In the model, a few simplifications were made which may cause inconsiderable effects on the results. A circular shape was used for the nut and bolt head. Furthermore, the washers were merged to the bolt head and nut because of having the same material elastic properties [24].

**Figure 2:**
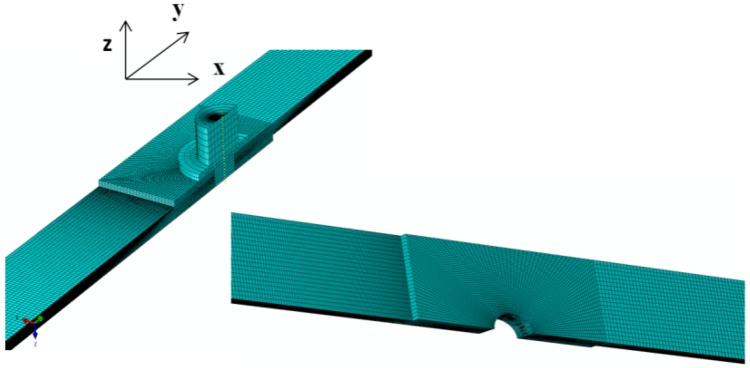
Finite element model of the bolted joints, same mesh density was used for the double bolt joints.

One-quarter of the full model, as shown in Figure 2, was used for the finite element analysis since the geometry and loading conditions of the joint model were completely symmetric with respect to XY and YZ planes. Symmetric displacement boundary conditions were applied to the nodes on the cut planes. Quadratic hexahedral elements (C3D8R) were used for meshing the model. As the vicinity of the bolt hole was the critical zone to be analysed, density of the mesh was appropriately refined in this region. Contact was implemented between the plates, bolt and inside the hole and washer and plates using a finite sliding formulation with a surface-to-surface discretization method. A Lagrange multiplier formulation was implemented, representing the contact pressure in a mixed formulation of pressure and contact surfaces. A penalty friction formulation was used with friction coefficients of 0.23, 0.35 and 0.25 between the plates, plates and the bolt shank and washer and top plate, respectively. The friction coefficient between the contacting parts was experimentally obtained from a test based on the sliding of a small piece of each part under its own weight on the sloped surface of the aluminium plate. It is noted that the friction coefficient may not be constant and can vary in the joint operating under mechanical loads [26,27,28]. However, as a simplification in this study, friction coefficients were considered to be constant during the loading process [4,24]. This was found to be an appropriate assumption for this study because of a good agreement achieved between the simulation and experimental results.

One end of the plates was constrained and the tensile load was applied to the other end. Bolt clamping force (preload) was applied using the Bolt Load option in ABAQUS. The amount of the bolt clamping force (*F_C_*) as a result of the tightening torque (15 N·m) was estimated to be 8.13 kN using Equation (1):

(1) $$F_{\text{c}}=\frac{\text{T}}{\text{K}.{\text{ d}}_{\text{bolt}}}=11811\text{ N}$$

where *T* is the tightening torque in N·m; *d_bolt_* is the bolt nominal diameter (6.35 mm); and *K* is the torque coefficient (0.205) [24]. Given several types of specimens used in the experimental and numerical investigations, a naming system was considered to define each configuration (Table 1). *W* and *e* are defined in Figure 1.

**Table 1:** Bolted joint specimens coding systems used in this work.

Parameters	SW1e1	SW1e2	SW1e3	DW1e1	DW1e2	DW1e3	SW2e1	SW2e2	SW2e3	DW2e1	DW2e2	DW2e3
W (mm)	25.4	25.4	25.4	25.4	25.4	25.4	30	30	30	30	30	30
e (mm)	9.5	12.7	15.875	9.5	12.7	15.875	9.5	12.7	15.875	9.5	12.7	15.875
Single bolt	✓	✓	✓				✓	✓	✓			
Double bolt				✓	✓	✓				✓	✓	✓

## 4. Verification of Finite Element Mode

**Figure 3:**
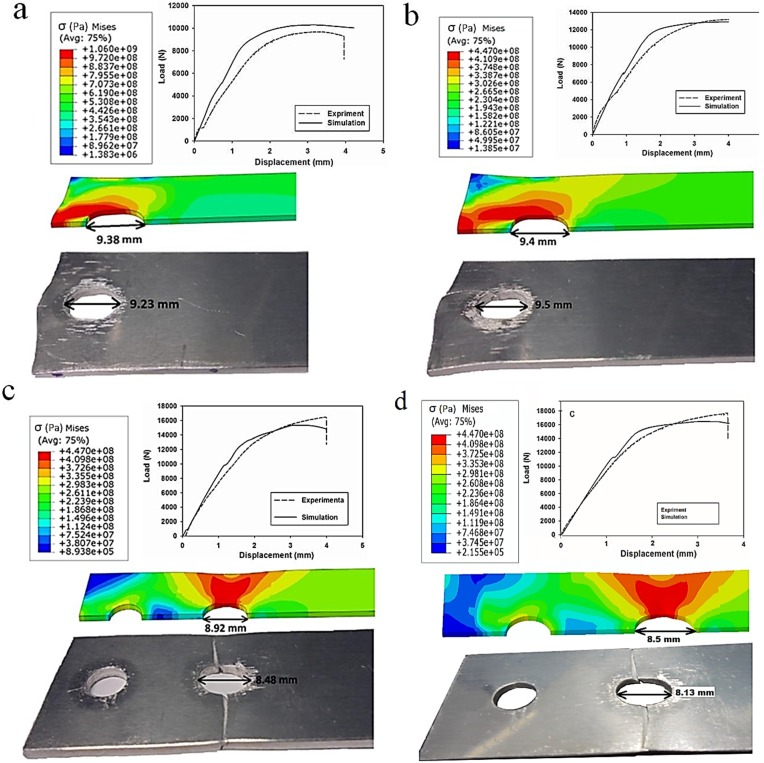
A comparison between experimental and numerical results of load-displacement curves along with the hole deformation: (**a**) SW1e1; (**b**) SW1e2; (**c**) DW1e1; and (**d**) DW1e2. The stress contours are von Mises stresses in Pa.

To validate the finite element modelling analysis, finite element models were generated with the same geometry and dimensions as the experimental specimens. Validations were accomplished by comparing the load-displacement data of the joint specimens obtained from the experimental and numerical methods until the displacement reached 4 mm. The results were in a very good agreement and the maximum discrepancy was found to be less than 6% in the maximum applied load. Additionally, the diameter of the holes after loading was compared for both simulations and experiments. The maximum discrepancy in the hole diameter was around only 5%. Generally, very good agreements observed between the experimental and numerical load-displacement curves along with the hole deformations (Figure 3) satisfactorily verified the finite element model and the numerical methodology to evaluate different geometric variables which will be discussed in the next section.

## 5. Results and Discussion

**Figure 4:**
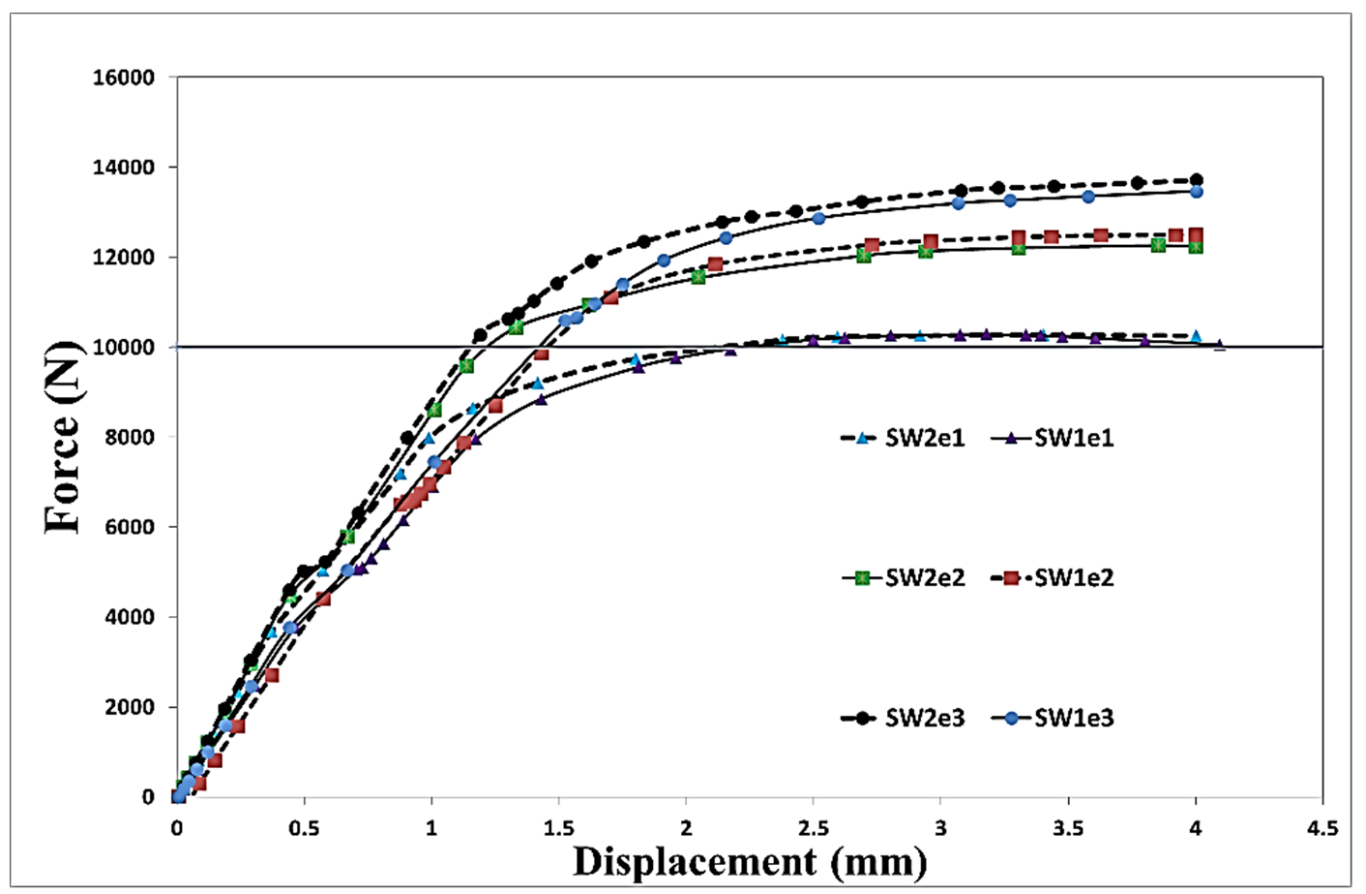
Numerical load-displacement curves of single bolt joints with different geometries.

**Figure 5:**
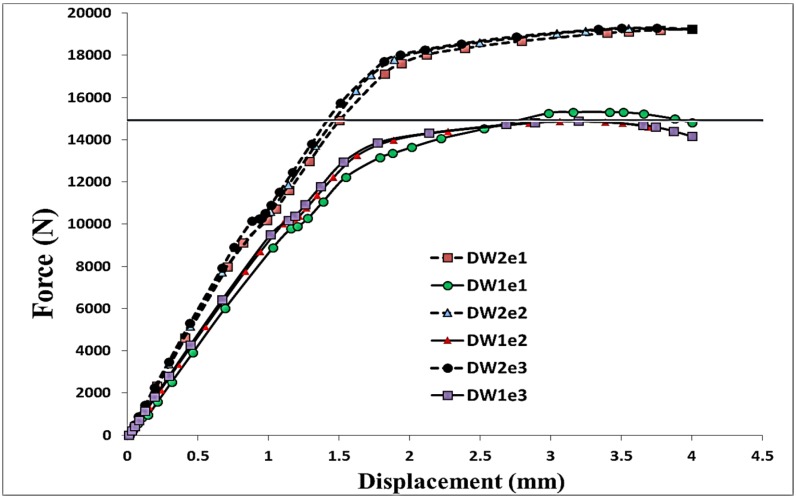
Numerical load-displacement curves of double bolt joints with different geometries.

Using the verified finite element modelling approach, 12 models with different configurations and dimensions, as listed in Table 1, were generated in order to investigate the effect of geometric parameters on the stress distributions within the bolted joint so as to predict the failure mode. The hole diameter, length and thickness of the plates were kept constant, whereas, two widths (*W*) and three edge distances (*e*) for both single and double bolt joints were considered as variables. In all models, an 11,811 N preload was applied as the first load step in order to firmly tighten the joint and clamp the aluminium plates. Subsequently, a longitudinal tensile displacement of 4 mm was applied to the end of the middle plate through a quasi-static process. In Figure 3, the load-displacement curves and local deformation of the hole obtained from the experiments and simulations are compared. For the experiments, the average values of three tests were presented. The standard deviation of the maximum load in SW1e1, SW1e2, DW1e1 and DW1e2 specimens were found to be 23 N, 19 N, 13 N and 16 N, respectively. As can be seen in this figure, shear-out was the failure mode in the single bolt joints while in the double bolt junctions, net-tension was found as the mode of failure. Figure 4 and Figure 5 show the load-displacement curves of single and double bolt joints of different specimens. Generally, it was understood that to reach the same displacement, double bolt junctions needed a greater tensile force. Moreover, from these two figures, the maximum load for each joint specimen causing a 4 mm elongation in the middle plate of the joint can be identified. DW1e1 in comparison with SW1e1 needed a higher force (by 49%) to reach the 4 mm displacement. This difference was 40% between DW3e3 and SW3e3. This revealed that the double bolt junctions had greater load carrying capacities than single bolt joints. Because of the different failure modes, the location of the crack initiation was totally different in single and double bolt junctions.

Figure 4 and Figure 5 show the effect of a change in width (*W*) and edge distance (*e*) on the behaviour of the load-displacement curves. From Figure 4, it can be understood that an increase in the width of the single bolt joints had no significant effect on the behaviour of the junction under tensile loading, whereas, with increasing *e,* the strength of the junction considerably enhanced. With increasing e from 9.5 mm (1.5*d*) to 12.7 mm (2*d*) and 15.9 mm (2.5*d*), the maximum load corresponding to the 4 mm displacement increased by 23% and 37%, respectively (*d* is the diameter of the bolt). On the other hand, in the double bolt joints, a change in *e* had almost no effect on the behaviour of the junction. But, increasing *W* from 25.4 to 30 mm enhanced the maximum load by 28% (Figure 5).

**Figure 6:**
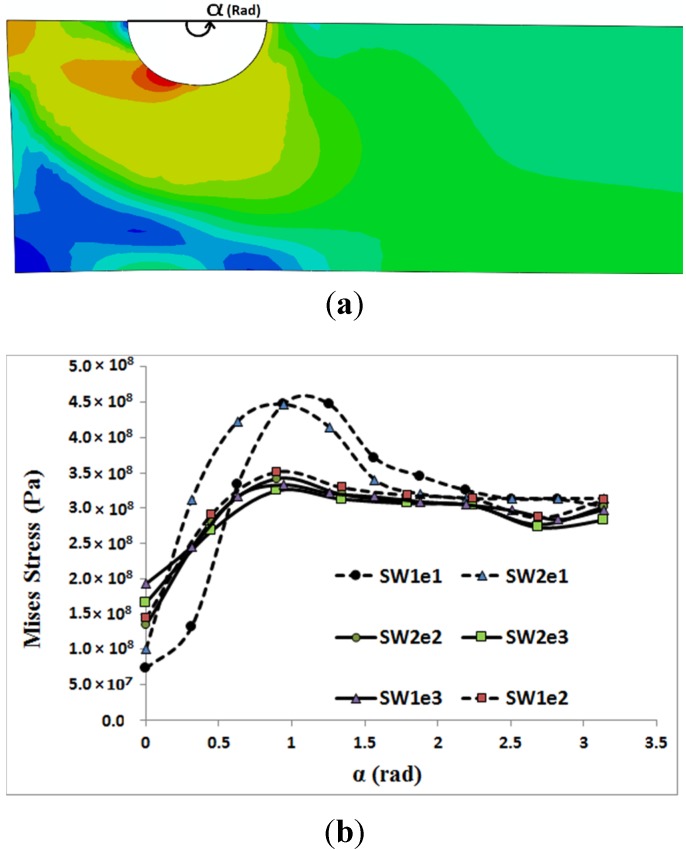
Von Mises stress distribution on the hole circumference *versus* angle α, when the tensile load in the middle plate of single bolt joints reached 10 kN: (**a**) stress contour; (**b**) stress profiles.

**Figure 7:**
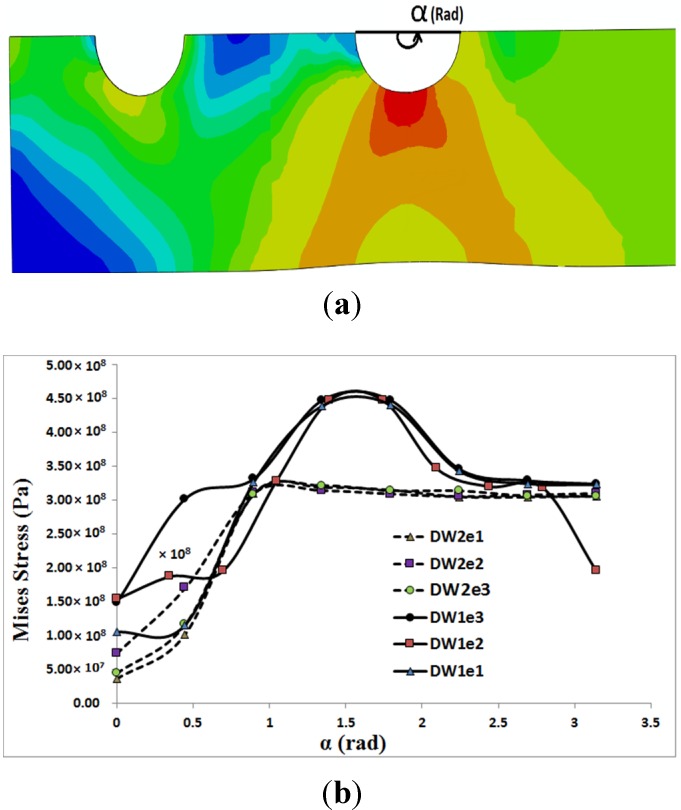
Von Mises stress distribution on the hole circumference *versus* angle α, when the tensile load in the middle plate of double bolt joints reached 14.9 kN: (**a**) stress contour; (**b**) stress profiles.

In Figure 6 and Figure 7, the distribution of von Mises stress on the hole circumference is plotted when the tensile load reached 10 kN in the single bolt joints and 14.9 kN in the double bolt joints, respectively. The line of tensile loads 10 kN and 14.9 kN is specified in Figure 4 and Figure 5. It can be realised from Figure 6 and Figure 7 that the initiation of cracks in the double bolt junctions was approximately at 2π/7 rad (90°), while in the single bolt joints, this initiation occurred at 2π/7 rad (approximately 50°). This was because of the difference in failure modes observed in the single and double bolt joints in experiments. In the single bolt junctions, for specimens with e = 9.5, with increasing force to 10 kN, shear-out failure mode occurred. This observation was confirmed with stress results shown in Figure 6. In SW1e1 and SW2e1 specimens, the maximum stress at the critical point reached almost the ultimate strength of the aluminium plate. While in the other specimens under the same load, the stress, even at the critical point, was far from the ultimate strength, but met the yield strength of the aluminium plate. For example, with increasing the load to 10 kN, the hole diameter of SW1e2 increased from 6.35 to 7.02 mm, confirming the occurrence of bearing failure mode.

**Figure 8:**
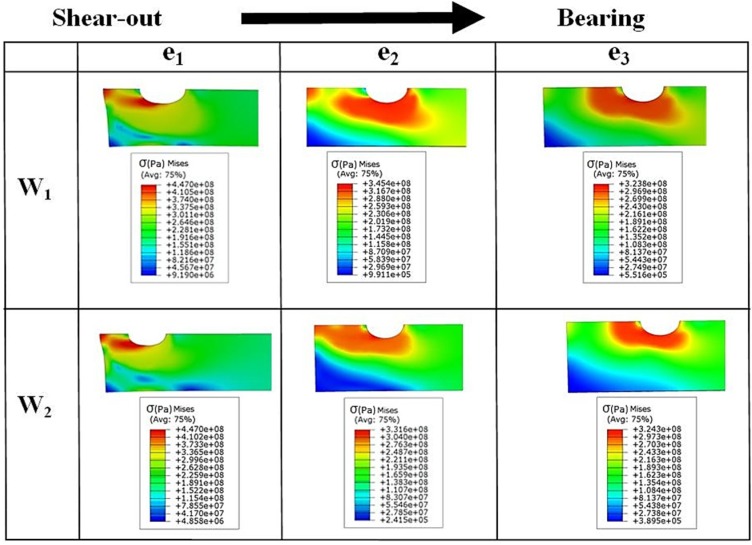
Von Mises stress (Pa) contours in the middle plate of single bolt joint specimens with different widths (*W_1_* and *W_2_*) and edge distances (*e_1_*, *e_2_*, and *e_3_*).

**Figure 9:**
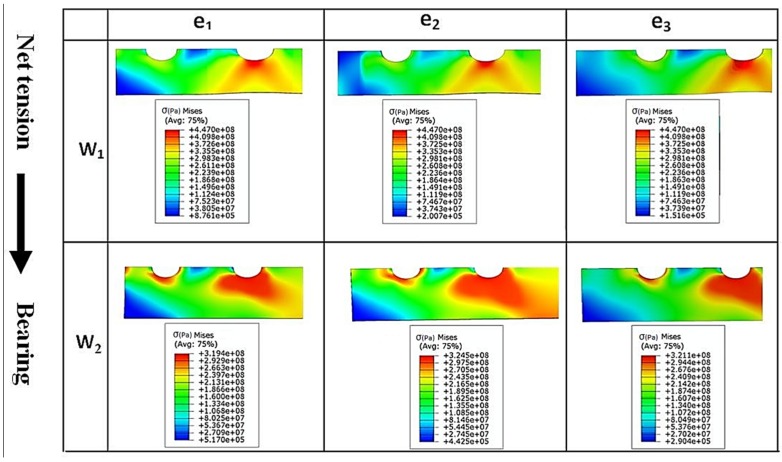
Von Mises stress (Pa) contours in the middle plate of double bolt joint specimens with different widths (*W_1_* and *W_2_*) and edge distances (*e_1_*, *e_2_*, and *e_3_*).

Based on the experimental test results and finite element stress results, net-tension was found as the catastrophic failure mode for the double bolt joints (Figure 3). It can be understood from Figure 5 that in the double bolt joints of DW1e1, DW1e2 and DW1e3 (smaller W), 14.9 kN was the maximum load required for these specimens to experience the net-tension failure mode (when the applied tensile load reached 14.9 kN, the net-tension failure occurred). In support of this, Figure 7 also shows that the von Mises stress at the critical point of the hole in the afore-mentioned specimens met the ultimate tensile strength of the aluminium plate. Whereas, the specimens with a bigger W had only plastic deformations. For instance, with increasing load to 14.9 kN, the hole diameter of DW2e1 increased from 6.35 to 6.91 mm. Thus, the bearing failure mode occurred in these specimens. Figure 8 and Figure 9 show the von Mises stress contours in the middle plate of all the single and double bolt joints, respectively. In Figure 8, it can be found that in the single bolt joints, by increasing the *e* parameter, the failure mode shifted from shear-out to bearing. On the other hand, Figure 9 shows that increasing the width of the plate in the double bolt joints shifted the failure mode from net-tension to bearing.

**Figure 10:**
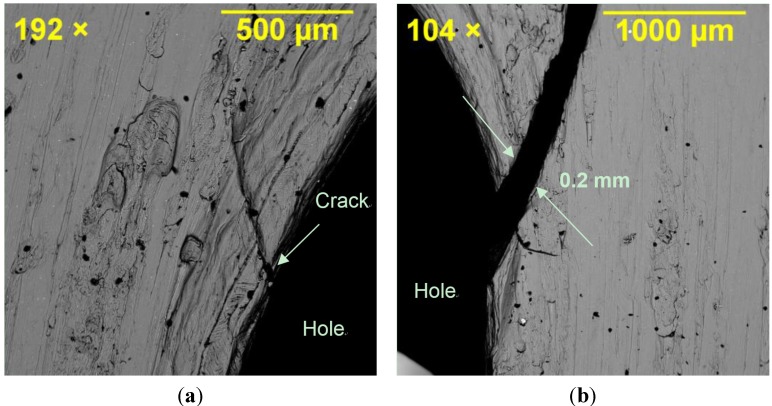
Crack opening at the shear-out region of the hole in SW1e1 specimen: (**a**) left side of the hole; and (**b**) right side of the hole.

**Figure 11:**
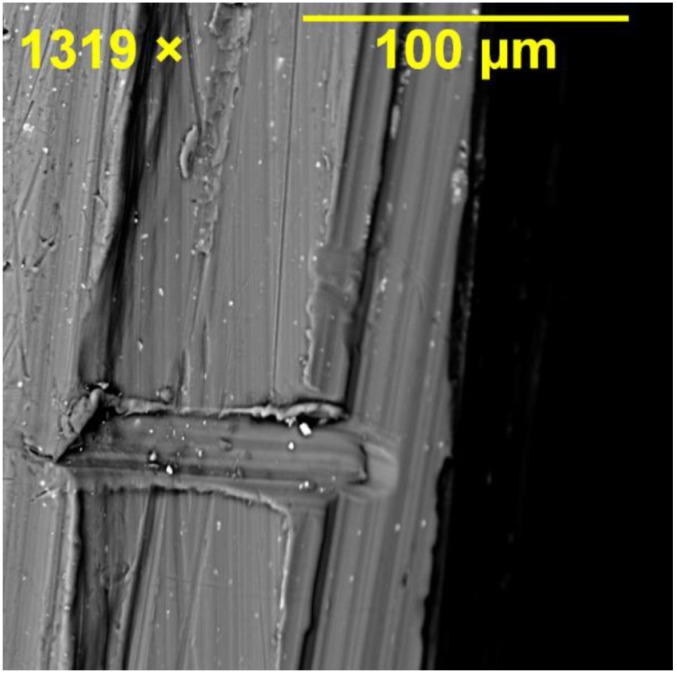
Shearing edge formed on the material surface of the cracked region in the middle plate of SW1e1 specimen.

Fractography of the failed specimens, particularly the middle plate, clearly showed the origin of the failure mode in different joint specimens depending on their geometry. Figure 10 shows SEM images of the hole region in SW1e1. Crack opening at the shear-out region of the hole can be seen where the main crack opened by approximately 0.2 mm due to the localised stress of 450 MPa (refer to Figure 8 for SW1e1) at the edge of the hole under tensile loading. Because of the symmetry, the hole had cracks in its both left and right sides verifying the symmetric finite element model in terms of loading, geometry and failure cracks. The shearing edge on the material surface of the cracked region is captured in Figure 11 where the vertical lines represent the shearing path that the material created as fracture occurred. Minor horizontal scratches can be seen which were caused in the removal of the cracked section.

**Figure 12:**
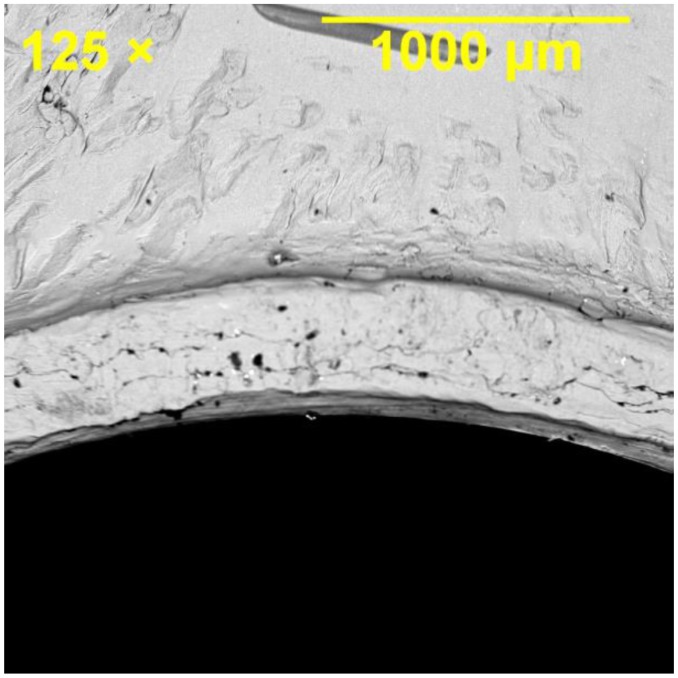
Bearing damage in the hole of the middle plate in SW1e3 specimen, for which bearing failure mode was dominant.

**Figure 13:**
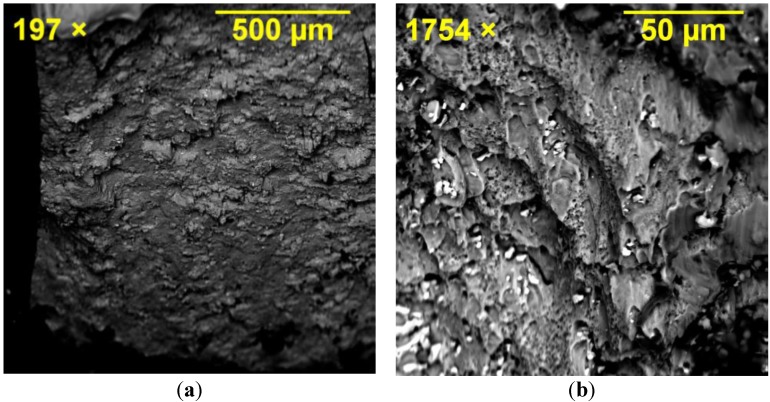
Fracture surface of the middle plate in DW1e1 showing a ductile dimple rupture after failure: (**a**) lower magnification; (**b**) higher magnification.

Bearing damage was inspected in the joint specimens failed through the bearing failure mode. Significant bearing deformation and surface damage were observed in the hole, as illustrated in Figure 12, where bruising of the material was evident as a result of bearing prior to the joint failure. Fracture surface of the middle plate in all the joint specimens (single and double bolt) exhibited ductile fracture features because of the ductility of Al 2024-T3 under tensile loading. The examination of the fracture surface of the specimens confirmed that the fracture initiated in the centre of the section followed by development of cracks and large shear stresses on 45° planes forming shear-lip regions. Ductile dimples and the growth of fracture by ductile dimple rupture can be seen in Figure 13.

## 6. Conclusions

A three-dimensional finite element model was developed to predict the tensile behaviour of Al 2024-T3 double-lap bolted joints with single and double fasteners. The model was verified by experimental tests and there was a good agreement between the experimental and numerical results.

- Shear-out was the dominant failure mode in the single bolt joints, while the net-tension failure mode occurred mainly in the double bolt junctions.
- The investigation revealed that the double bolt junctions offer a greater load carrying capacity under tensile loading when compared to single bolt joints. They resist loads 40%–49% magnitude higher.
- The initiation of failure in the double bolt junctions occurred at the critical edge of the hole where there is a high stress concentration (π/2 rad); while in the single bolt joints the initiation of failure occurred at 2π/7 rad.
- In the single bolt joints, geometric parameter of width (*W*) showed no significant effect on the tensile behaviour of the joint, whereas, with an increase in the edge distance (*e*), the strength of the joint increased considerably.
- In the double bolt joints, changing *e* had almost no effect on the tensile behaviour of the joint. However, increasing *W* from 25.4 to 30 mm increased the load carrying capacity by 28%.
- In the single bolt joints, increasing the edge distance (*e*), shifted the failure mode from shear-out to bearing favourably.
- Increasing width of the plate in the double bolt joints beneficially shifted the failure mode from catastrophic net-tension to bearing.
